# Evaluation and Treatment of Cardiac Tamponade in a Pregnant Patient

**DOI:** 10.1155/2020/8703980

**Published:** 2020-01-15

**Authors:** Matthew P. Romagano, Krunal Patel, Shauna Williams, Joseph J. Apuzzio

**Affiliations:** Rutgers New Jersey Medical School, Newark, New Jersey, USA

## Abstract

Cardiac tamponade is an uncommon but life-threatening emergency that may occur in pregnant women. There is a plethora of causes, but prompt diagnosis and intervention is imperative to optimize both maternal and fetal outcomes. We report on a case of a large pericardial effusion leading to cardiac tamponade occurring in the 32^nd^ week of gestation in a previously healthy woman. Rapid recognition and a multidisciplinary team meeting resulted in a therapeutic pericardial window and drainage and relief of symptoms. The woman underwent an uncomplicated repeat cesarean delivery at term with a positive neonatal outcome. This case highlights the importance of a rapid diagnosis and a team-based approach to managing a complex medical condition like cardiac tamponade in pregnancy.

## 1. Introduction

Cardiac tamponade is a life-threatening emergency requiring prompt diagnosis and treatment. Patients often present acutely with chest pain, dyspnea, and tachypnea with cardiovascular collapse, but the disease course may be subacute or chronic with an initial asymptomatic period followed by progression [1]. It can be difficult to discern whether common findings in pregnancy like peripheral edema, dyspnea, and hyperventilation are physiologic or pathologic in etiology. In this article, we describe a case of a pregnant woman presenting with a large pericardial effusion and cardiac tamponade, who was treated with an emergent pericardial window and drainage.

## 2. Case

**Figure 1:**
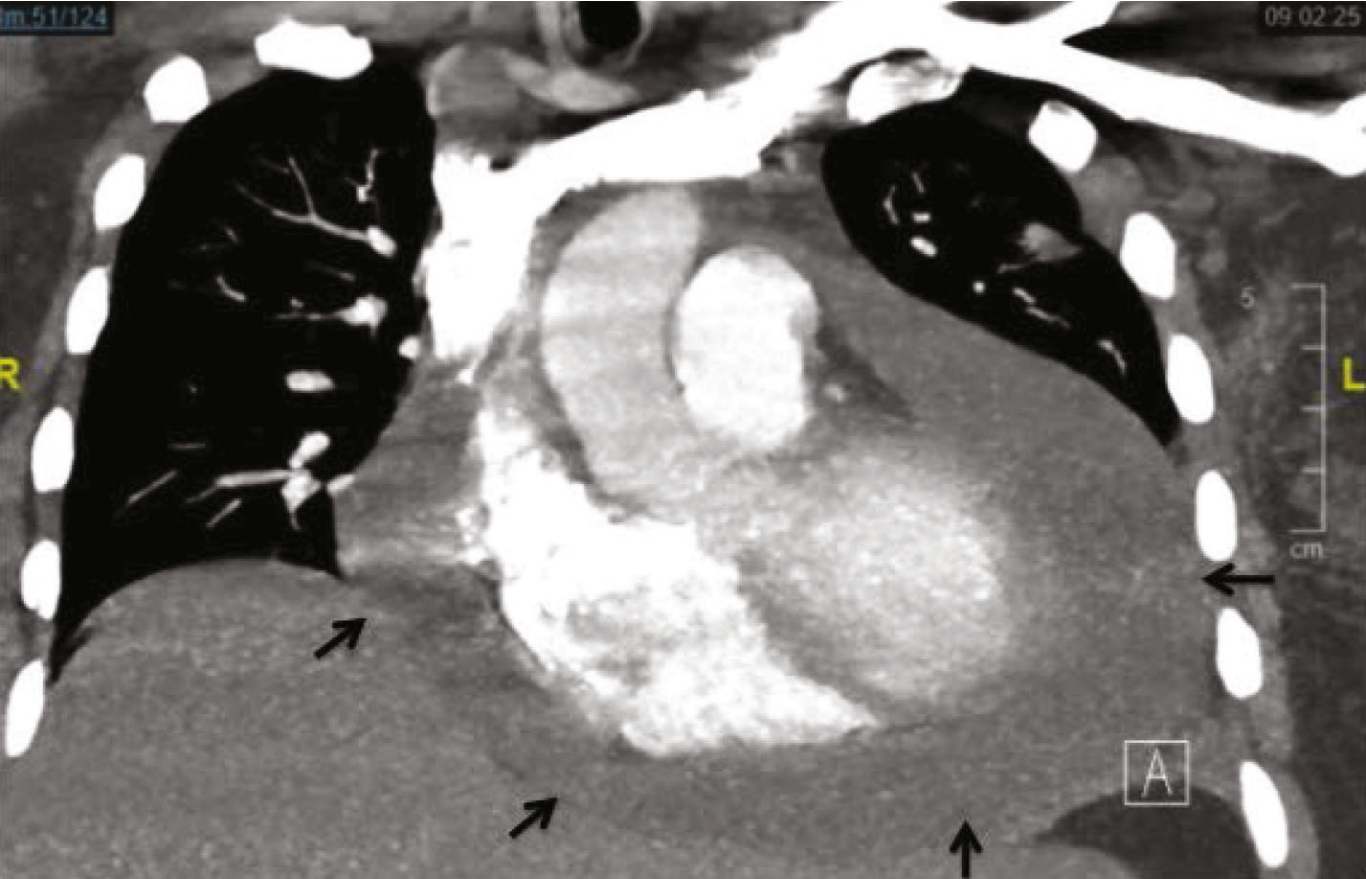
Computerized tomography demonstrating large pericardial effusion (arrows).

A 29-year-old Hispanic gravida 3, para 2-0-0-2 at 32 weeks of gestation presented to an outside hospital with a 3-day history of worsening chest pain, dyspnea, orthopnea, and shoulder pain. She was previously in good health without comorbid medical issues. Initial work-up at the outside hospital included a computed tomography (CT), which was significant for a large pericardial effusion with no evidence of pulmonary embolism (Figure 1). She was transferred to our institution for further management.

On arrival, the patient was uncomfortable and leaning forward, with a blood pressure of 127/80 mmHg, pulse of 124 beats per minute, respirations of 24 times per minute, and an oxygen saturation of 97%. She appeared in distress. There was tachycardia and distant heart sounds on physical examination, but no jugular venous distension noted. Laboratory evaluation revealed no significant abnormalities. Arterial blood gas analysis demonstrated a mixed metabolic and respiratory acidosis. Cardiac ischemia markers were negative. An electrocardiogram (ECG) demonstrated sinus tachycardia and electrical alternans.

**Figure 2:**
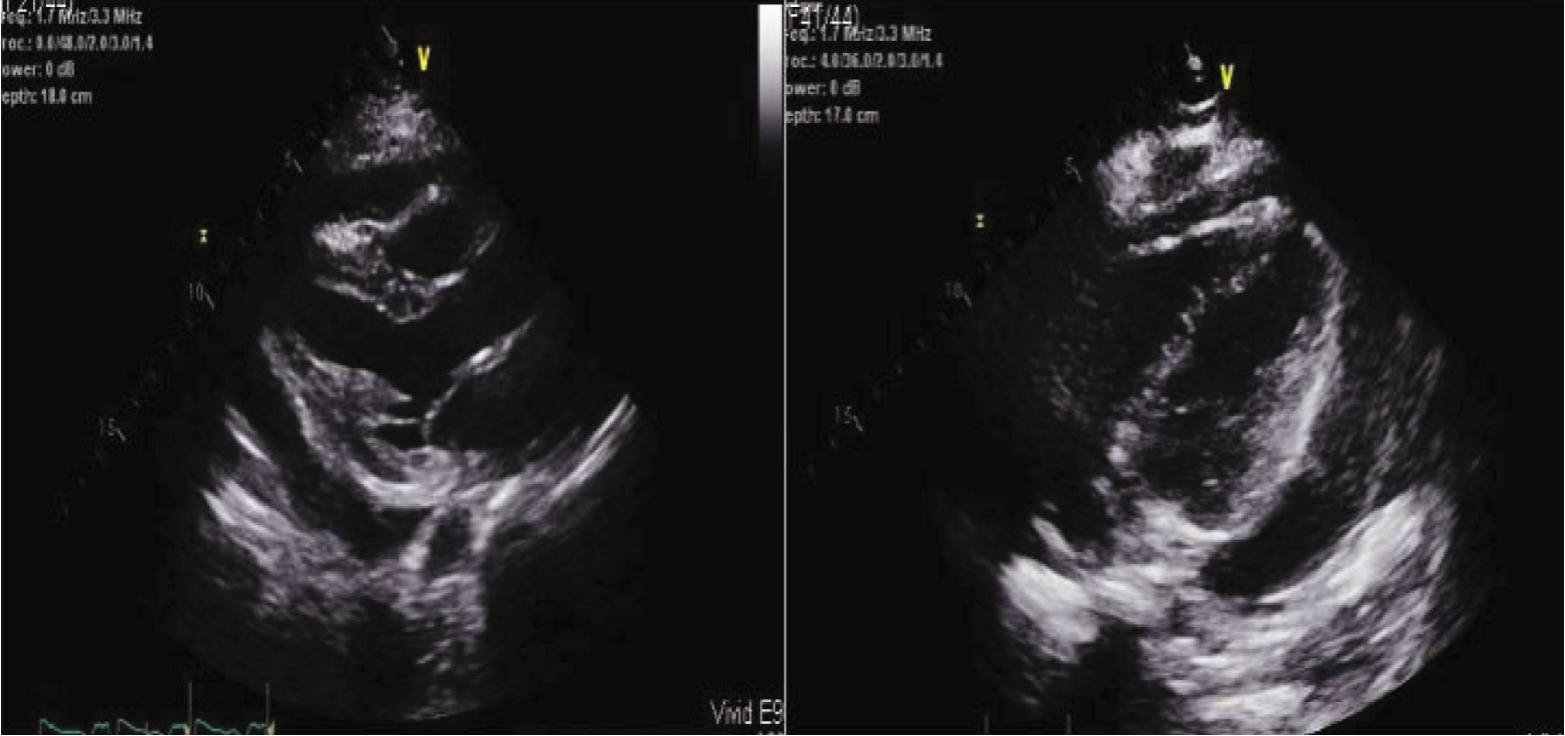
Echocardiogram images demonstrating a large pericardial effusion. Cardiac tamponade physiology was present during the study.

A transthoracic echocardiogram showed a large pericardial effusion with tamponade physiology and a left ventricular ejection fraction of 50% (Figure 2). The patient had worsening chest pain and discomfort. The obstetric team performed fetal monitoring which demonstrated no abnormalities. A multidisciplinary team met at the patient's bedside and consisted of maternal fetal medicine (MFM), cardiothoracic surgery, neonatology, cardiac anesthesia, and cardiology specialists.

It was decided that surgical pericardial window and drainage with drain placement was the most appropriate treatment because the majority of the pericardial effusion was posterior and would not be amenable to percutaneous drainage. The patient received a dose of betamethasone for fetal benefit, and underwent an uncomplicated pericardial window and drainage under general anesthesia with standard monitoring and continuous fetal monitoring. A total of 400 mL of serous fluid was drained from the pericardial space. Obstetric nursing, neonatology, and MFM team members were present in case emergent cesarean delivery was required. The fetus had periods of minimal heart rate variability and intermittent late decelerations, but this improved at the conclusion of the procedure. Her vital signs normalized and symptoms resolved postoperatively. The pericardial drain was removed on the day of discharge, postoperative day 7.

Maternal blood laboratory studies provided no obvious etiology. The pericardial fluid cultures and cytology were negative for tuberculosis, bacterial or fungal infection, or malignancy. Serum autoimmune and collagen vascular disease work-up was negative. Thyroid function tests were normal. Serum antibody titers were positive for Coxsackie B virus infection.

The patient had an uneventful postoperative course following pericardial window and drainage and returned at 38 weeks for scheduled repeat cesarean delivery and bilateral tubal ligation. A repeat echocardiogram prior to surgery revealed no reaccumulation of pericardial fluid. The cesarean was uncomplicated, and she gave birth to a live male infant weighing 3420 grams with Apgars of 9 and 9 at 1 and 5 minutes, respectively. She was discharged home on postoperative day 3. Her postpartum course was uncomplicated.

## 3. Discussion

This case demonstrates the importance of prompt evaluation, work-up, and treatment of suspected cardiac tamponade in pregnancy. A multidisciplinary team successfully managed this case jointly. This approach led to positive maternal and neonatal outcomes.

Cardiac tamponade is caused by compression of the heart chambers and can rapidly lead to death. It involves the accumulation of fluid, blood, gas, or other substances in the pericardial space leading to cardiac compression, impaired diastolic filling, and a reduction in stroke volume and cardiac output [2]. In 1935, Beck described cardiac tamponade as a classic triad of hypotension, increased jugular venous pressure, and quiet heart sounds [3]. It is characterized by tachycardia, decreased voltage on ECG, and pulsus paradoxus. Pulsus paradoxus is a decrease in systolic blood pressure greater than 10 mmHg on inspiration, and is strongly associated with tamponade [4]. Tamponade physiology is demonstrated on echocardiography by abnormal posterior motion of the right ventricular free wall during diastole [5]. An enlarged cardiac silhouette may be seen on chest X-ray (CXR) [1]. Confirmation of the diagnosis can only be made based on hemodynamic and clinical response to drainage of the effusion.

Etiologies of pericardial effusion and tamponade include infectious, iatrogenic, trauma, malignancy, collagen vascular disease, aortic dissection, and other causes. Echocardiogram is considered the first-line imaging modality [1]. Further work-up should include a complete blood count, chemistry panel, thyroid function, CXR, ECG, antinuclear antibody, and pericardial fluid analysis and biopsy. The pericardial fluid should be sent for Gram stain, bacterial and fungal cultures, cytology, acid-fast bacilli stain, and mycobacterial culture. Evaluation for common viral etiologies may also be considered such as coxsackievirus, cytomegalovirus, HIV, and parvovirus B19 [6]. In pregnancy, testing for these viruses is relevant because of the risk of congenital infection. In our case, Coxsackie B maternal serum titers were suggestive of, but not diagnostic for, maternal infection. Coxsackie B virus is associated with myocarditis but is also a documented cause of pericardial effusion [7]. Treatment of tamponade involves drainage of the pericardial effusion via percutaneous pericardiocentesis or surgical drainage via a pericardial window.

Pericardial effusion leading to cardiac tamponade is a relatively rare entity in pregnancy. A review of the literature yields several case reports of cardiac tamponade associated with various pregnancy and non-pregnancy-related conditions. It has been described in preeclampsia and hemolysis, elevated liver enzymes and low platelets (HELLP) syndrome [8, 9]. In one case, a woman diagnosed with HELLP syndrome developed a subcapsular liver hematoma resulting in extrapericardial cardiac tamponade, which was treated conservatively with a positive maternal outcome [8]. In another case, a pregnant woman presented with dyspnea and a pericardial effusion with tamponade treated with pericardiocentesis. No cause was identified at the time. She was subsequently diagnosed with angiosarcoma 6 months after delivery after she again developed dyspnea, and a right atrial mass was discovered [10]. Another case was associated with a large anterior mediastinal mass found to be a B-cell lymphoma treated with pericardial drainage and chemotherapy [11]. Other cases of tamponade due to aortic abscess [12], scleroderma [13], and iatrogenic injury [14] have been described. The current case demonstrates the rarity but seriousness of cardiac tamponade and the importance of quick intervention.

Cardiopulmonary disease is the leading cause of maternal death in the United States [15]. The physiologic changes of pregnancy predispose women to an increased incidence of peripheral edema, dyspnea, and hyperventilation, which can mimic cardiac disease. However, as the current case suggests, prompt evaluation of the gravida presenting with chest pain, dyspnea, peripheral edema, and changes in vital signs is essential, as significant and life-threatening cardiac disease may be the underlying etiology. A multidisciplinary approach to care is paramount for positive maternal and fetal outcomes.
